# Difference in Intestine Content of *Caenorhabditis elegans* When Fed on Non-Pathogenic or Pathogenic Bacteria

**DOI:** 10.3390/mi14071386

**Published:** 2023-07-07

**Authors:** Farzad Rezaeianaran, Martin A. M. Gijs

**Affiliations:** Laboratory of Microsystems, Ecole Polytechnique Fédérale de Lausanne, CH-1015 Lausanne, Switzerland; martin.gijs@epfl.ch

**Keywords:** *C. elegans*, high-resolution imaging and analysis, bacterial load, microfluidics

## Abstract

We investigated the bacterial food digestion and accumulation in wild-type adult *Caenorhabditis elegans* (*C. elegans*) worms that have fed on either non-pathogenic RFP-expressing *Escherichia coli* (*E. coli*) OP50 or pathogenic-RFP-expressing *Pseudomonas aeruginosa* (*P. aeruginosa*) PAO1 during the first 4 days of adulthood. Once the worms had completed their planned feeding cycles, they were loaded on microfluidic chips, where they were fixed to allow high-resolution z-stack fluorescence imaging of their intestines utilizing a Spinning Disk Confocal Microscope (SDCM) equipped with a high-resolution oil-immersion objective (60×). IMARIS software was used to visualize and analyze the obtained images, resulting in the production of three-dimensional constructs of the intestinal bacterial load. We discovered two distinct patterns for the bacteria-derived fluorescence signal in the intestine: (i) individual fluorescent spots, originating from intact bacteria, were present in the fluorescent *E. coli*-OP50-fed worms, and (ii) individual fluorescent spots (originating from intact bacteria) were dispersed in large regions of diffuse fluorescence (RDF), originating from disrupted bacteria, in fluorescent *P. aeruginosa*-PAO1-fed worms. We performed a semi-automated single-worm-resolution quantitative analysis of the intestinal bacterial load, which showed that the intestinal bacterial load generally increases with age of the worms, but more rapidly for the fluorescent *P. aeruginosa*-PAO1-fed worms.

## 1. Introduction

Host–microbiota interactions have a profound and wide-ranging impact on the health, aging and diseases in humans [1,2,3,4,5]. The microbiota can affect the development and the health of central nervous systems [6,7], possibly play a causal role in diseases such as diabetes and obesity [8], mediate drug action [9] and fight against toxins and pathogens in the intestine [5,9]. However, these studies are mostly based on correlations, as discovering the underlying mechanisms through which the gut microbiota exerts such an influence is extremely complicated due to the diversity of human genomes and the bacterial species present in the intestine [1,10].

The *C. elegans* nematode is a genetically tractable model organism for studying host–microbiota interactions and, in particular, bacterial pathogenesis, innate immunity and aging [11]. As the genome of this nematode bears considerable similarity to the genome of humans, the discoveries made using this organism are also relevant to health and diseases in humans [11,12,13]. The millimeter-sized transparent organism relies on the consumption of bacteria for its development and reproduction [8,9]. In laboratories, the nematode is often maintained on Nematode Growth Medium (NGM) plates containing a single type of bacteria (mostly *E. coli*) [14]. The relationship between the worm and *E. coli* is dynamic and changes over time. In the larval stages, the bacteria are thought to be simply a food source, which are killed by the pharyngeal grinder and, thus, no live bacteria in principle reach the intestine. In young adult worms, few intact bacteria manage to pass through the pharyngeal grinder without being damaged and form a community in some areas in the intestine and thus establish a commensal relationship with the worm [9]. As the worm ages, however, the decline in the effectiveness of the pharyngeal grinder in killing bacteria, innate immunity, ingestion and defecation leads to excessive proliferation of bacteria, which can become mildly pathogenic to the worm [9,15]. 

The interaction between the bacteria and the worm is often characterized in terms of TD_50_ (the time after which 50% of the worms feeding on a bacterial lawn are dead, obtained from survival/lifespan curves), the average number of colony-forming bacteria per worm at a given time point, and the gene expression of the worm that is measured either through fluorescence microscopy, blotting techniques or quantitative real-time polymerase chain reaction (qRT-PCR) analysis. We can use TD_50_ as a simple way to compare the pathogenicity of different bacteria assuming that the considered bacteria support the worm adequately from a nutritional standpoint. For example, an adult wild-type worm population feeding on pathogenic *P. aeruginosa* PAO1 has TD_50_ = 2.84 days [16], while the same population fed on *E. coli* OP50 has TD_50_ = 12.93 days [15]. Both of these bacteria have been shown to provide adequate nutrition to the worm, but also cause its demise through intestinal colonization [14,16,17]. However, the much shorter lifespan of worms fed *P. aeruginosa* PAO1 shows that this bacterium exhibits a higher degree of pathogenicity compared to *E. coli* OP50. Despite the extensive research on non-pathogenic *E. coli* OP50 [15,17,18,19] and pathogenic *P. aeruginosa* [16,20,21,22,23,24,25,26,27,28], which reveals and discusses the innate immunity pathways of the worm and the virulence mechanism of pathogenic *P. aeruginosa*, little is known about the impact of the bacterial food source on the digestion/absorption of bacterial material by the worm through its intestine. 

The use of microfluidics for studying *C. elegans* was reported for the first time in 2007 [29] and, since then, the field has grown considerably and devices for different purposes have been proposed [30,31,32,33,34]. The main advantages of microfluidics are the ease in handling large worm populations and the reversible immobilization that enables high-resolution imaging [31]. Generally speaking, microfluidic devices can either enable high-throughput studies at low imaging resolution [30,35] or low-throughput studies at a high imaging resolution [36,37,38,39,40,41,42]. The vast majority of host–microbiota studies have been and are still carried out using conventional techniques and, despite the great potential of microfluidic devices, there are very few microfluidic devices that are designed for such studies. 

The first microfluidic device for implementing a *C. elegans* infection assay was proposed by Yang et al. [43] in 2013. The device comprised 32 chambers that were divided into four groups. In each group, different doses of a given antimicrobial compound could be administered to the 8 chambers through concentration gradient generators. Worm populations were infected on-chip with *Staphylococcus aureus* and, afterward, the efficacies of the antibiotic compounds as a function of their doses were assessed through survival curves. As a result, this device is mostly suitable for in vivo antibiotic compound screening. Lee et al. [44] reported a microfluidic device for performing *C. elegans* killing assays. The worm and the pathogen of interest could be loaded in a large chamber, where the motility and survival of the worms and the expression of *irg-1::GFP* could be monitored in vivo through low-resolution imaging. However, due to the lack of compartments, the identities of the worms could not be tracked over time. 

Viri et al. [45] reported a microfluidic device for in vivo study of the bacterial transit in *C. elegans*. The device featured four separate lanes each of which comprised five compartments. A total of 1 to 3 worms were loaded into each compartment where they were fed fluorescent-tagged bacteria. Through low-resolution fluorescence imaging and subsequent image analysis, the spatiotemporal evolution of intestinal bacterial load of the freely moving worms could be obtained. In this way, the periodic behavior associated with ingestion of bacteria and its clearance from the intestine was monitored and its temporal periodicity was determined. The worms could also be immobilized in the tapered structures present in the compartment, which rendered them incapable of feeding. Afterward, low-resolution fluorescence images were acquired from these worms to obtain the time constant of the bacterial transition. In a subsequent work, Viri et al. [46] improved the design of their previous device [45] such that worms could be immobilized in individual traps for up 30 h while being able to feed on fluorescent bacteria. This device could replicate the previous results and the improved immobilization enabled relatively high-resolution fluorescence imaging. Thus, in the fluorescence images of the immobilized worms, the intact bacteria that appeared as individual fluorescent spots could be distinguished from the disrupted ones, which appeared as diffuse fluorescence. This allowed the observation of the digestion process and, as a result, the associated time constant was determined. It should be mentioned that in their work [46], they used a widefield microscope for fluorescence imaging. This inherently limits the imaging resolution, as the light signal is received from many focal planes including those that are out of focus. Consequently, despite their usage of a 50× objective for fluorescence imaging, the fluorescence signal from intact bacteria that lie in the out-of-focus planes will blur the image. This effect, in particular, is pronounced in areas of high bacterial density and ultimately poses an insurmountable obstacle for the correct determination of the source of an eventual diffuse fluorescence signal from the intestine (as the fluorescent signal originating from bacteria could originate from the diffuse-fluorescence of disrupted bacteria present in the plane of focus, or spot-like intact bacteria present in out-of-focus planes), and thus for distinguishing the intact bacteria from the disrupted ones.

SDCMs offer superior resolution compared to widefield microscopes by incorporating pinholes that block the light signal from out-of-focus planes. Therefore, to improve upon the previous works by Viri et al. [45,46], we aim to use SDCM for high-resolution z-stack fluorescence imaging and advanced image analysis techniques. This allows us to investigate bacterial food digestion and accumulation in *C. elegans* worms with an unprecedented resolution. To achieve this, we relied on loading the worm population of interest on a simple microfluidic chip where the worms could be subsequently fixed for high-resolution imaging. We preferred this approach to the more conventional method whereby previously anesthetized worms are mounted on an agarose pad before they are observed [47,48]. While anesthetics such as levamisole could be used to immobilize the worm, we are not sure if they would also stop the intestinal peristaltic motion. For our advanced image analysis to be accurate, even movements as little as 5–10 µm should be avoided, especially if they occur frequently and in a periodic manner. This is to prevent any unwanted alteration in the size and the intensity distribution of the detected bacterial spots. Alternatively, these movements can also lead to the appearance of new but illusory bacterial spots. While it is also possible to mount previously fixed worms on agarose pads, we preferred to carry out the fixation procedure and imaging on a microfluidic chip. This allows us to fix and image all the worms at the same time and also shows that our approach can be applied to any existing microfluidic device for host–microbiota studies.

## 2. Materials and Methods

### 2.1. Materials and Chemicals

Polydimethylsiloxane (PDMS) Sylgard 184 (Dow^®^), 4-inch 550 µm Si wafers, 5-inch Chromium/soda–lime glass masks and trimethylchlorosilane (TMCS) (Sigma-Aldrich, St. Louis, MO, USA) were acquired from the Center of MicroNanoTechnology (CMi) at EPFL (Lausanne, Switzerland). Kayaku Advanced Materials (KAM) SU-8 3050 was bought from Micro Resist Technology GmbH (Berlin, Germany). Glass coverslips of 45 mm × 70 mm × 170 µm were obtained from Biosystems Switzerland AG (Muttenz, Switzerland). Saint-Gobain Tygon™ ND 100–80 Tubing (inner diameter and thickness of 0.02 inch and outer diameter of 0.06 inch) was purchased from Fisher Scientific (Reinach, Switzerland). NGM plates were ordered from the Solution Preparation Facility at EPFL. In order to prepare S-basal, 5.85 g of NaCl, 6 g of KH_2_PO_4_ and 1 g of K_2_HPO_4_ were first dissolved in H_2_O until a final volume of 1 L was realized. This solution was subsequently sterilized via autoclaving and, afterward, 1 mL of cholesterol solution (5 mg mL^−1^ in ethanol) was added using a sterile technique to obtain S-basal. In order to make S-medium, 500 µL of 1 M potassium citrate (pH 6), 500 µL of trace metals solution, 150 µL of 1 M CaCl_2_ and 150 µL of 1 M MgSO_4_ were added using a sterile technique to 50 mL of S-basal. The materials required for the preparation of S-basal, S-medium and lysogeny broth (LB) for bacteria culture and tetracycline were purchased from Sigma-Aldrich Chemie GmbH (Buchs, Switzerland). The Laboratoire des Polymères (LP) at EPFL (Lausanne, Switzerland) kindly provided 4% paraformaldehyde (PFA) solution in phosphate-buffered saline (PBS), which was used for the fixation of *C. elegans*.

### 2.2. Worm and Bacteria Culture and Preparation

We used *C. elegans* wild-type (WT) Bristol N2 strain in our experiments. The worm populations were synchronized by suspending gravid adult worms (that can have slightly different ages) overnight inside a 50 mL falcon tube containing 8 mL of S-medium. During this time, due to the lack of food, the L1s that hatch from the eggs laid by the adults underwent developmental arrest [49] and, thus, a synchronized L1 population was obtained on the next day. Afterward, the dead adults that naturally settle at the bottom of the falcon tube were removed via aspiration and the remaining synchronized L1 population was transferred to multiple 1.5 mL Eppendorf tubes to be centrifuged for 4 min at 2000 RPM. About 100 L1s were aspirated from the bottom of one of the Eppendorf tubes and were spread on NGM plates seeded with *E. coli* OP50 and maintained there for 46 h at 22 °C until they became adults and were thus ready for the experiments. 

*E. coli* OP50 was cultured overnight in LB at 37 °C on a shaker. Red Fluorescent Protein (RFP)-labeled *E. coli* OP50 was kindly provided by the *C. elegans* Ageing Laboratory at the University College London. Plasmid *pRZT3::dsRED*, which also contained genes for tetracycline resistance, was used to transform *E. coli OP50* and produce the RFP-labeled *E. coli* OP50. RFP-labeled *E. coli* OP50 was cultured in LB containing 10 µg/mL of tetracycline overnight at 37 °C on a shaker. RFP-labeled *P. aeruginosa* PAO1, which was kindly provided by the Laboratoire des Polymères (LP), constitutively express mCherry (*attTn7::miniTn7T2.1-Gm-GW::PA1/04/03::mCherry*) in the bacterial cytoplasm. RFP-labeled *P. aeruginosa* PAO1 was cultured overnight in LB at 37 °C on a shaker.

### 2.3. Fabrication of the Microfluidic Chip

The microfluidic chip was designed in Clewin 4.0 (WieWeb software, Hengelo, The Netherlands) and through standard mask fabrication techniques, and the designed layout was then transferred to a chromium/soda-lime glass mask. Next, the mask was used to create a 75 µm high SU-8 mold on a Si wafer using standard soft photolithography techniques. To facilitate the separation of the polydimethylsiloxane (PDMS) chip from the SU-8 mold, the mold was treated with trimethylchlorosilane (TMCS) and, afterward, PDMS with a base-to-curing agent ratio of 10:1 was cast onto the mold and cured for 2 h at 80 °C inside an oven. Once the PDMS cured and cooled down, it was cut according to the design of the device and the resulting PDMS block was removed from the mold. Afterward, using a biopsy punch, inlets and outlets were made in the PDMS block. The PDMS block and a glass coverslip measuring 45 mm × 70 mm × 170 µm were bonded together, utilizing oxygen plasma surface activation, to create the microfluidic device. The microfluidic chip was then placed on a hotplate for 10 min at 80 °C to improve the bonding strength.

### 2.4. Image Processing and Statistical Analysis

Raw z-stack fluorescence images were processed in IMARIS (version 9.9.1). The software constructs three-dimensional (3D) images from z-stack fluorescence images, which can be further analyzed using the built-in toolkits. The results of image analysis by IMARIS are exported to excel files that are processed in an automated fashion in MATLAB software (version 2022a). The processing involves selecting the data of interest and performing basic arithmetic operations on them. The processed data can then be easily imported in GraphPad Prism software (version 9.5.0) to be plotted and tested for statistical significance. The one-tailed Mann–Whitney test was utilized to establish statistical significance.

### 2.5. Microscopy Platform and the Imaging Parameters

We utilized two different microscopes in our experiments. A Zeiss Axio Imager.M2 operated in brightfield mode and equipped with a 2.5× Zeiss objective was used for preparing the microfluidic chip, loading worms on the chip and their subsequent fixing. High-resolution z-stack fluorescence imaging was realized using Visitron’s CSU-W1—a spinning disk confocal microscope equipped with (i) two laser light sources for fluorescence imaging in the RFP and the Green Fluorescent Protein (GFP) channels, running at wavelengths 561 nm and 488 nm, respectively; (ii) an Olympus U PLAN S APO 60×/1.42 NA oil immersion objective; (iii) emission filter cubes ET605/70m and ET525/50m, by Chroma Technology (Rockingham, VT, USA), for the RFP and the GFP channels, respectively; and (iv) a Hamamatsu ImagEMX2 electron-multiplying CCD (EMCCD) camera. The imaging was carried out with the following parameters: exposure time of 60 ms, laser power for both the RFP and the GFP channels of 50%, gain of the EMCCD camera set to 200, pinhole size of 50 µm and z-stack imaging in a 50 µm range with a step size of 0.2 µm.

## 3. Experimental

### 3.1. The Design and Operation of the Microfluidic Chip Platform

**Figure 1:**
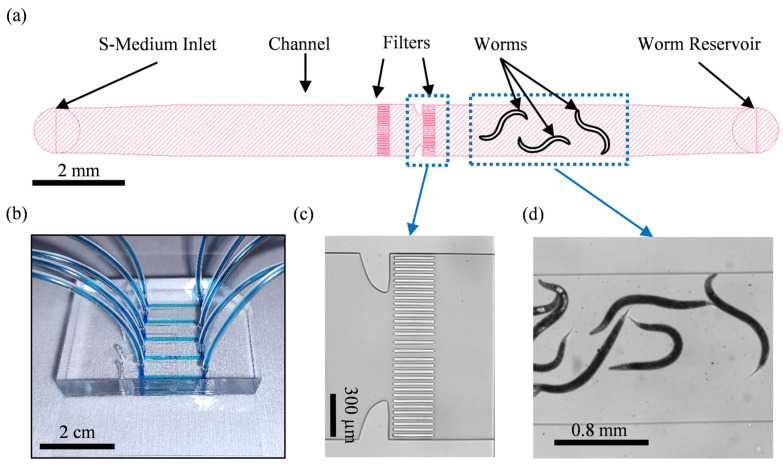
Microfluidic chip for high-resolution imaging of bacteria in the intestine of *C. elegans*. (**a**) The schematic shows the typical design of the lanes in the device. Every device has 5 lanes that are 1300 µm wide and 75 µm high. The worms are contained in the channel by the filters. (**b**) Photograph of a fabricated microfluidic device. The tubing and the channels of the device are filled with a blue dye for better visibility. (**c**) Brightfield image showing the filter structures. (**d**) Brightfield image showing the worms that are loaded in the microfluidic chip.

Our microfluidic chip consists of five identical lanes, the layout of which can be seen in Figure 1a. Our device allows on-chip fixation and subsequent high-resolution imaging of adult worms. 

The lanes are 2 cm long, 1300 µm wide and 75 µm high, the last of which makes the device capable of accommodating adult worms. Furthermore, each lane features two filter structures whose functions are to confine the loaded worms to the right side of the channel and provide control over the number of worms present on the chip. An example of a fabricated microfluidic device is shown in Figure 1b. The filter structure, which is shown with more detail in Figure 1c, consists of rows of 20 µm-wide PDMS slabs that are spaced 15 µm apart except in the middle where the spacing is 30 µm. The spacings have been chosen such that even upon the application of relatively high flow rates, which results in adult worms being compressed against the filter structures, no adult worm passes through the filter structures. 

The microfluidic chip is operated while being observed in the brightfield mode using a Zeiss Axio Imager.M2 equipped with a 2.5× Zeiss objective. Before worms can be loaded on the chip to be fixed and imaged, a chip initialization step should take place. Therefore, S-medium is first injected from the inlet to the channels using a neMESYS syringe pump (CETONI GmbH, Korbußen, Germany). However, as the air in the channels is being replaced by S-medium, some bubbles of air are bound to form. To eliminate these, the outlet tubing is clipped and the channel is pressurized by injecting 10 µL of S-medium through the inlet. The increased pressure promotes the outward diffusion of air through PDMS and thus the channel is cleared from air bubbles within 15–20 min. Afterward, the outlet tubing is unclipped and is placed inside an Eppendorf tube containing the adult worms of interest suspended in S-medium. The worms can then be loaded on the chip (Figure 1d) by using the neMESYS syringe pump to aspirate the S-medium with a flow rate of 500 nL s^−^1. Once around 10–15 worms are present in the channel, an Eppendorf tube containing a 4% PFA fixation solution is placed at the outlet and, by aspirating with a flow rate of 80 nL s^−1^, the fixation solution is introduced in the channel. The worms become fixed by staying in contact with the fixation solution for about 5 to 10 min, after which the inlet and outlet tubings are cut and fastened and the microfluidic chip can be taken to an SDCM for high-resolution z-stack fluorescence imaging. 

### 3.2. Experimental Planning

**Figure 2:**
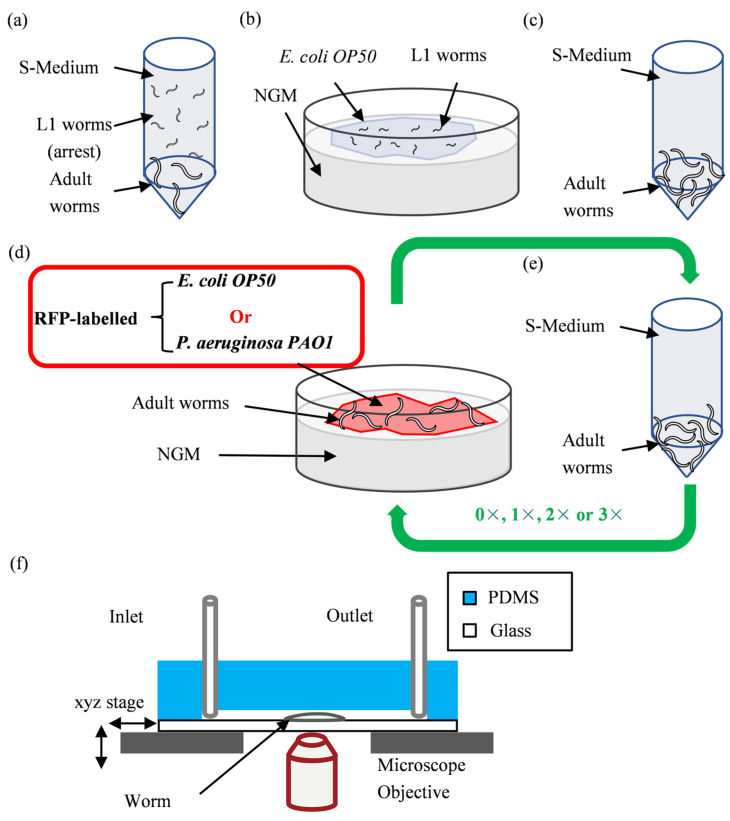
Protocol and experimental planning for high-resolution imaging of fluorescent *E.coli* and *P. aeruginosa* in the intestine of *C. elegans*. (**a**) An adult population of worms are suspended overnight in S-medium to obtain a multitude of synchronized L1s in developmental arrest the following day. (**b**) About 100 synchronized L1s are placed on an NGM plate seeded with *E. coli* OP50 and grown there for 46 h at 22 °C until they reach adulthood. (**c**) Using S-medium, the adult worms are removed from the NGM plate and are placed inside a 1.5 mL Eppendorf tube, where they naturally settle at its bottom. This allows the removal of *E. coli* OP50 through the replacement of the supernatant with fresh S-medium (dilution of 1:10). This procedure is repeated 10 times (1:10^10^ total dilution) to drastically reduce the number of *E. coli* OP50 present. (**d**) The adults are then fed for 24 h by being placed on an NGM plate that, depending on the experiment to be performed, is either seeded with RFP-labeled *E. coli* OP50 or RFP-labeled *P. aeruginosa* PAO1. (**e**) To prevent the progenies from overcrowding the plates and mixing with the adult population, every 24 h, the adults along with their progenies are washed off the plate using S-medium and are placed inside a 1.5 mL Eppendorf tube. As the L1 progenies remain suspended in S-medium, similar to “step c”, a 1:10^10^ dilution is carried out to remove them and the remaining adults are then moved to a new NGM plate seeded with the same bacteria as before (e.g., if the adult worms were feeding on RFP-labeled *E. coli* OP50, the new NGM plate is also seeded with this bacteria). Steps d and e are repeated according to the number of days the adult worms are planned to be fed on either RFP-labeled *E. coli* OP50 or RFP-labeled *P. aeruginosa* PAO1 (0×, 1×, 2× and 3× of repetitions for 1 day, 2 days, 3 days and 4 days of feeding, respectively). After the last iteration of “step e”, the worms remain for an hour within the Eppendorf tube containing S-medium to ensure the complete digestion of recently eaten bacteria. (**f**) The worms are then transferred to the microfluidic device where they are fixed using a 4% PFA solution. Shortly after, the tubings are cut and fastened with clips and the microfluidic device is moved to a SDCM, where high-resolution imaging of the intestinal bacteria is carried out.

We wish to study the bacterial food digestion and accumulation in wild-type adult *C. elegans* worms that have fed on either RFP-labeled *E. coli* OP50 or *P. aeruginosa* PAO1 during the first 4 days of adulthood via high-resolution z-stack fluorescence imaging (Figure 2). First, we synchronize the worm population (Figure 2a) by suspending an adult worm population in a falcon tube containing S-medium overnight, during which the adult worms lay eggs. The next day, a large number of synchronized L1s will be present in the falcon tube since the lack of food causes the hatched larvae to undergo developmental arrest [49]. About 100 of these synchronized L1s are then transferred to an NGM plate seeded with *E. coli* OP50 where they will be kept for 46 h at 22 °C until the they reach adulthood (Figure 2b). At this point, the adult worms can be fed either the RFP-labeled *E. coli* OP50 or the RFP-labeled *P. aeruginosa* PAO1. However, when the adult worm population is to be taken to a NGM plate seeded with either one of these two fluorescent bacteria, it must be ensured that ideally no unlabeled *E. coli* OP50 is transferred with it. Therefore, after adult worms are washed off the NGM plate using S-medium, they are placed inside a 1.5 mL Eppendorf tube where they naturally sink to its bottom. This facilitates the gradual elimination of *E. coli* OP50 through the replacement of the supernatant with fresh S-medium (dilution of 1:10). This procedure is repeated 10 times (1:10^10^ total dilution) and thus the number of *E. coli* OP50 present in S-medium is significantly reduced (Figure 2c). The adult worms can now be safely placed on NGM plates seeded with either RFP-labeled *E. coli* OP50 or *P. aeruginosa* PAO1 to feed for 1, 2, 3 and 4 days (Figure 2d). As high-resolution imaging at each time point of interest (i.e., after 3 days of feeding on RFP-labeled *E. coli* OP50) requires fixation, the same worms cannot be studied at different time points. Additionally, while the adult worms are feeding (on either of the fluorescent bacteria), they also lay eggs that hatch into L1s. 

These L1s not only mix with the adult population, which later can become a source of confusion, but they can also rapidly deplete the NGM plate of bacterial food. Therefore, every 24 h, the adult worms are separated from L1s through serial dilutions (Figure 2e), which is carried out in a manner similar to Figure 2c with a total dilution of 1:10^10^. Afterward, the adult worms are transferred to a fresh NGM plate (Figure 2d) seeded with the same bacteria as before (e.g., if the adult worms were feeding on RFP-labeled *E. coli* OP50, the new NGM plate is also seeded with this bacteria). It follows naturally that the steps described in Figure 2d,e should be repeated in a cyclic manner depending on the number of days the worms are to feed on fluorescent bacteria. This translates to 0×, 1×, 2× and 3× of repetitions for 1 day, 2 days, 3 days and 4 days of feeding, respectively, with 0× meaning that the steps described in Figure 2d,e are performed once. After the step in Figure 2e is carried out for the last time, the worms should remain suspended in S-medium (Figure 2e) and thus be starved for 1 h before their intestines can be imaged. As the starvation period of 1 h is considerably longer than the 70s to 90 s time interval between bacterial ingestion and intestinal clearance in wild-type adult worms (70 to 90 s as reported by Viri et al. [45] and 60 to 110 s as reported by Ghafouri and McGhee [50]), it is safe to assume that any recently ingested non-pathogenic bacterium has the possibility to be completely digested before fluorescence imaging is carried out. Therefore, any bacteria that will still be imaged via fluorescence microscopy can be thought to have at least a persistent presence in the gut if not colonizing it. When the worms have starved for an hour, they are loaded on the microfluidic chip and subsequently undergo the fixation procedure (Figure 2f). Afterward, the tubings are cut and fastened and the microfluidic chip is taken to an SCDM for high-resolution z-stack fluorescence imaging. Regarding the fixation of RFP-labeled *P. aeruginosa* PAO1, it should be mentioned that the lab (Laboratoire des Polymères, EPFL) that provided us this bacterium used a 4% PFA solution as per their fixation protocol and hence, in good faith, we follow the same protocol.

### 3.3. Imaging Procedure and Analysis

**Figure 3:**
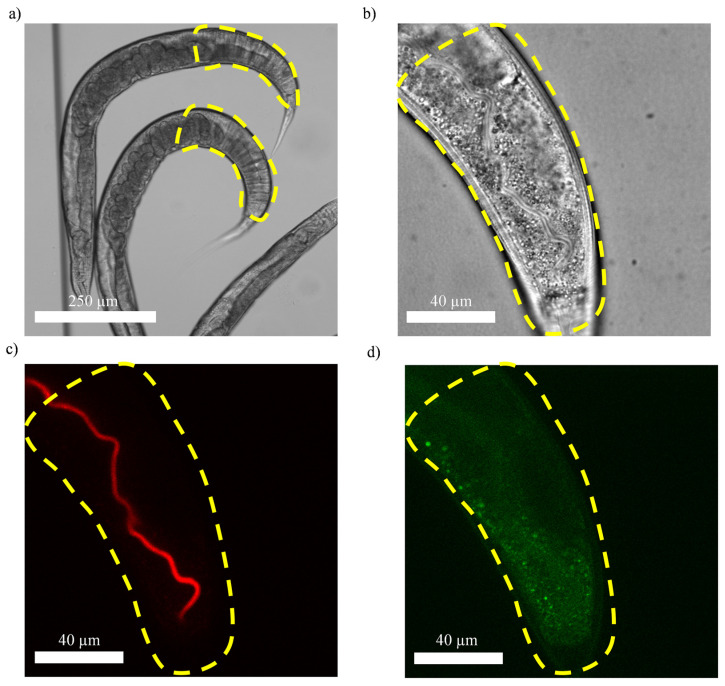
Imaging of representative fixed worms that had RFP-expressing *P. aeruginosa* PAO1 as their food source (duration of feeding is 1 day in (**a**) and 2 days in (**b**–**d**)). (**a**) Brightfield image of the fixed worms where the hindgut, as the key area for high-resolution imaging, is marked. High-resolution (**b**) brightfield image, (**c**) RFP channel image showing the diffuse fluorescence originating from the disrupted bacteria and (**d**) GFP channel image showing the worm’s autofluorescence. Lack of overlap in the fluorescence signal in (**c**,**d**) indicates that fluorescence in the RFP channel is not due to the worm’s autofluorescence r ((**a**): 10× objective; (**b**–**d**): High-resolution imaging using an oil-immersive 60× objective).

We start the imaging procedure with a low-magnification brightfield image (Figure 3a) to first locate the hindguts of the worms. We avoid imaging the worms that are overlapping or whose hindguts are merely touching each other. In the former case, the fluorescence signal of the two overlapping worms cannot be simply differentiated. In the latter case, when one of the worms is being imaged, the other worm that is very close to it is also being exposed and is thus subjected to potential fluorophore photobleaching [51]. Thus, the worm that is imaged second will always have its fluorescence signal intensity skewed toward a lower value.

Once the hindguts of the worms of interest are identified, we carry out high-resolution imaging of the hindguts in the brightfield mode and the RFP and the GFP channels, an example of which is shown in Figure 3b, 3c and 3d, respectively. Figure 3b is a brightfield image of the hindgut used to set the field of view more accurately and to determine the center of focus for the z-stack fluorescence imaging. Afterward, we perform z-stack fluorescence imaging in the RFP channel (Figure 3c) to observe the fluorescence signal originating from either disrupted (the case in Figure 3c) or intact bacteria. The parameters used for the fluorescence imaging are previously discussed in the “Microscopy Platform and the Imaging Parameters” section. We optimize these parameters to have a decent signal-to-noise ratio, minimize the fluorophore photobleaching and avoid the saturation of the fluorescence signal in the camera. Furthermore, we choose a sufficiently large range of focus (50 µm) to image the intestine across its height. However, since the 60× objective used for high-resolution imaging affords a limited field of view (140 µm × 140 µm), the hindgut needs to be imaged twice. Thus, we first image the part of hindgut that is closest to the tail of the worm and afterward image the other part that lies further away from the tail.

The adult wild-type worms exhibit autofluorescence, which at least partly originates from the intracellular-lysosome-derived granules within the intestinal cells [52,53].

This autofluorescence signal can be observed in the Blue Fluorescent Protein (BFP), GFP and RFP channel [52]. The exact chemical makeup for the fluorescent species responsible for the autofluorescence is not known [52]. It has been reported that the spatial overlap between the autofluorescence in the GFP and RFP channel is limited [52]. This suggests that the fluorescent species responsible for the GFP and RFP signal may be biologically distinct. Based on our observations, the autofluorescence signal originating from the RFP species (in the intestine of the worm) is negligible compared to the bacteria-derived RFP signal. However, in some of our observations, the autofluorescence signal originating from the GFP species (in the intestine of the worm) could also appear in the images acquired in the RFP channel (as suggested by the total spatial overlap of the signal in the RFP and the GFP channel). An example of the autofluorescence in the GFP channel in the hindgut of an adult worm is shown in Figure 3d. The purpose of these types of images is to ensure that the fluorescence signal observed in the RFP channel (Figure 3c) is not due to autofluorescence of GFP species present in the intestine of the worm (Figure 3d). In the event that an overlap between fluorescence signals in the RFP and GFP channel is observed, the fluorescence signal in the RFP channel should not be attributed to the presence of either intact or disrupted bacteria.

**Figure 4:**
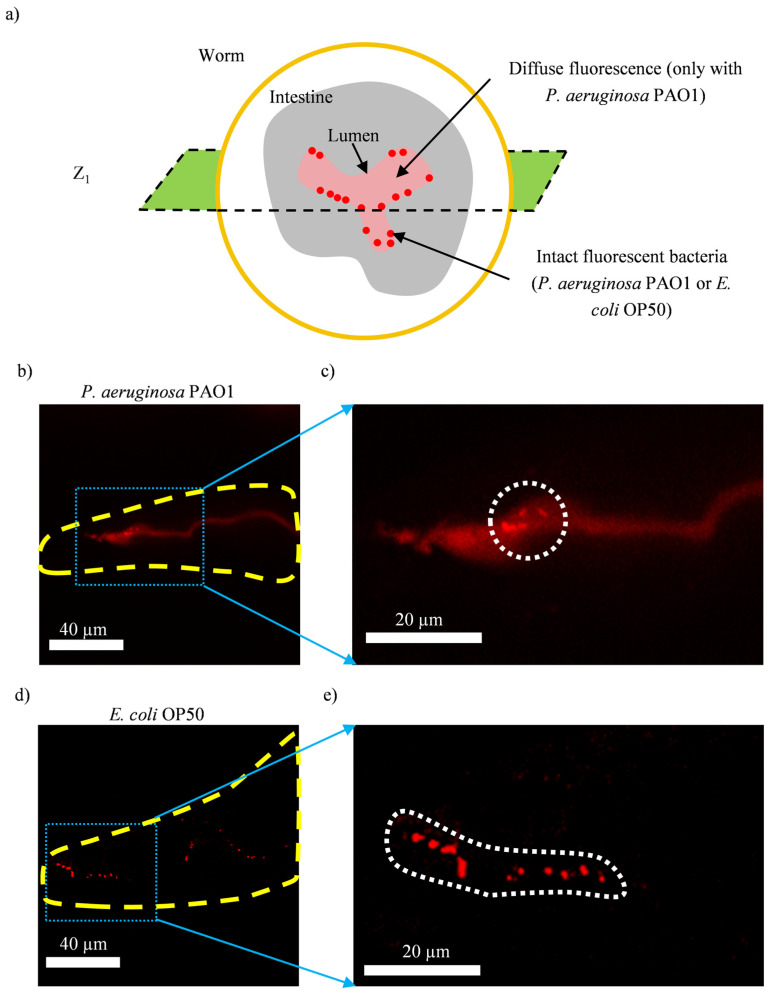
High-resolution z-stack fluorescence imaging (in the RFP channel) of representative fixed worms that had RFP-labeled bacteria (*P. aeruginosa* PAO1 or *E. coli* OP50) as their food source for 4 days. (**a**) An illustration of the worm cross-section where individual fluorescent spots, originating from intact fluorescent bacteria (*P. aeruginosa* PAO1 or *E. coli* OP50), and diffuse fluorescence, originating from disrupted bacteria, that is present only in fluorescent *P. aeruginosa-*PAO1-fed worms, can be observed in one of the slices (Z_1_). Z-stack imaging is required to capture all fluorescence signals across the height of the intestine. Example slices from the z-stack images (in the RFP channel) of worms that have fed on fluorescent *P. aeruginosa* PAO1 (**b**) and *E. coli* OP50 (**d**). (**c**) Magnified view of the blue rectangle in “b”, which shows diffuse fluorescence and individual fluorescent spots (marked by the white circle) in fluorescent *P. aeruginosa*-PAO1-fed worms. The presence of diffuse fluorescence from disrupted *P. aeruginosa* PAO1 does not exclude the presence of intact *P. aeruginosa* PAO1. (**e**) Magnified view of the blue rectangle in “d”, in which individual fluorescent spots (delineated by the white dashed line) originating from intact fluorescent *E. coli* OP50 can be clearly observed.

High-resolution z-stack fluorescence images of the intestine of the worms fed on either RFP-expressing *E. coli* OP50 or *P. aeruginosa* PAO1 reveal two distinct patterns for the bacteria-related fluorescence signal (Figure 4a). In the case of RFP-labeled *P. aeruginosa-*PAO-fed worms, diffuse fluorescence originating from disrupted bacteria and individual fluorescent spots originating from intact bacteria can be observed (Figure 4b,c). It should be emphasized that the presence of diffuse fluorescence from disrupted *P. aeruginosa* PAO1 does not rule out the presence of intact *P. aeruginosa* PAO1, and that the fluorescence signal from the latter can eventually be obscured by the fluorescence signal from the former. In marked contrast, in the case of RFP-labeled *E. coli*-OP50-fed worms, only individual fluorescent spots originating from intact bacteria can be seen (Figure 4d,e).

**Figure 5:**
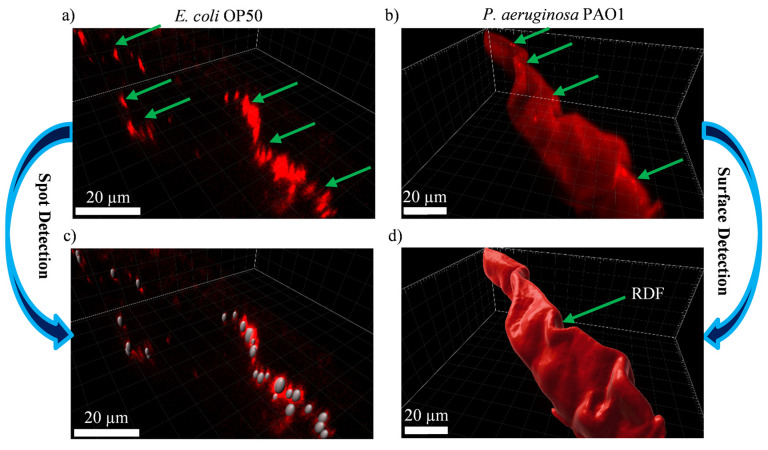
Visualization and analysis of the raw z-stack fluorescence images in IMARIS. Three-dimensional images of representative worms fed on either fluorescent *E. coli* OP50 (for 3 days) or fluorescent *P. aeruginosa* PAO1 (for 2 days) are constructed by assembling all the slices of the z-stack fluorescence images in the RFP channel. (**a**) Individual fluorescence spots (as shown by green arrows) originating from intact bacteria spread through the intestine of RFP-labeled *E. coli-*OP50-fed worms. (**b**) Individual fluorescent spots dispersed (as shown by green arrows) in a region of diffuse fluorescence (RDF) in the intestine of fluorescent *P. aeruginosa-*PAO1-fed worms. As the pattern of the fluorescence signal is different in a and b, two different methods are used to analyze the bacterial load. (**c**) Spot detection is governed by an estimation of the smallest fluorescent spot size as well as the threshold value for the local background-corrected intensity at the center of the spots, which is referred to as the “Quality” parameter in IMARIS. We choose the value of the “Quality” parameter such that detected spots match the actual spots and false positives are not present. Once the spots are detected, their sizes still need to be determined through the IMARIS “local contrast” method in which only the region around the center of the spot that has an intensity higher than a certain threshold is considered to be part of the spot. It should be mentioned that the perceived spot size is affected by the perspective projection that is used to display the 3D image. (**d**) Surface detection in IMARIS is conducted by setting a threshold value for the fluorescence intensity that can then be used to define a surface. In our case, we set this value such that it is always 600 higher than the average background noise in each set of experiments. Afterward, using this surface, one can define the RDF as the volume within which the fluorescence intensity exceeds the threshold value.

We use IMARIS software to enhance the visualization and analysis of the raw z-stack fluorescence images. IMARIS constructs a 3D image by putting together all the slices belonging to the same channel, which in our case is the RFP channel. Figure 5a,b show representative examples of such 3D images. In agreement with our discussion earlier regarding Figure 4, in the fluorescent *E. coli*-OP50-fed worms, the pattern of the fluorescence signal takes the form of individual spots that are spread through the volume of the intestine (Figure 5a), while in the case of fluorescent *P. aeruginosa-*PAO1-fed worms, it takes the form of individual spots that are dispersed in a region with diffuse fluorescence (RDF) (Figure 5b). This observation prompts us to employ different methods for the analysis of the bacterial load between the fluorescent *E. coli*-OP50-fed worms and the fluorescent *P.* aeruginosa-PAO1-fed ones. In a previous work [54], we expounded the detection and analysis of fluorescent spots in IMARIS. As the procedure is applicable here, but only to the fluorescent *E. coli*-OP50-fed worms (Figure 5a), we also briefly explain it here. The fluorescent spot detection begins with providing an estimate for the smallest spot size. In our case, we use 1 µm in the “x” and the “y” directions and 2 µm in the “z” direction to account for the point spread function of the microscope. The smallest spot size together with an IMARIS internal parameter called “Quality”, which is related to the local background-corrected fluorescence intensity at the center of spots, control the detection of spots. If the “Quality” is set too low, it leads to false positives and, if set too high, it leads to false negatives. Therefore, the “Quality” parameter should be chosen such that the detected spots match the actual spots and, in our case, a value of 150 satisfies this requirement. The spot detection procedure also makes it possible to correct for the contribution of worm autofluorescence to bacterial load. It should be first emphasized that there is no spatial overlap between the autofluorescence signal and bacteria-derived signal. Additionally, for every spot that is detected in the RFP channel by IMARIS, we also check whether the same spot also appears in the GFP channel. If the spot is also present in the GFP channel, the spot is assumed to originate from the worm autofluorescence and is discarded from the analysis. At this point, the spots are detected; however, their sizes are yet to be determined through the “local contrast” method in IMARIS. In this method, a local background correction is applied to the intensity distribution around each spot. Afterward, only the region around the center of each spot that has an intensity higher than a certain threshold (local contrast threshold) is deemed to be part of that spot. Consequently, the threshold should be set (80.4 in our case) such that the sizes of the finalized detected spots match visually with those of the actual spots (Figure 5c). To ensure that the detection of spots and their sizes is consistent across all the fluorescent *E. coli*-OP50-fed worms, the values for the estimation of the smallest spot size, the “Quality” parameter and the local contrast threshold are kept the same for all the said worms. Once the procedure for the detection of fluorescent spots is completed, the volumes and the average intensities of the finalized detected spots can be exported to excel datasheets for the purpose of bacterial load analysis. 

The approach used for fluorescent *E. coli*-OP50-fed worms, however, is not applicable to the fluorescent *P.* aeruginosa-PAO1-fed worms, as the intensity of RDF is too high to be ignored. Additionally, as mentioned earlier, the fluorescence signal originating from the intact fluorescent *P. aeruginosa* PAO1 bacteria can be masked by the signal from the disrupted fluorescent *P. aeruginosa* PAO1 bacteria and, thus, a bacterial load analysis based on separating these two types of fluorescence signal will not be entirely meaningful. Consequently, we decide to consider the RDF in its entirety, including the fluorescent spots that are spread within it, as one object for analysis (Figure 5b). In IMARIS, this can be achieved by using the “Surface” functionality. A surface can be defined such that its fluorescence signal intensity is equal to a given threshold value. In our case, in each set of experiments, for example, for worms that have fed on RFP-labeled *P. aeruginosa* PAO1 for 1 day, we measure the background noise (measuring the intensity where no worm is present), which ranges from 2770 to 2900 across all the experiments. The threshold value used for defining the surface is set 600 higher than the background noise and thus ranges from 3370 to 3500.

This surface can be then used to define RDF as the volume within which the fluorescence intensity is higher than the threshold value (Figure 5d). Once the RDF, defined in this manner, is calculated by the software, its average intensity and volume can be extracted for bacterial load analysis. As the quantification of the bacterial load relies on measuring the volume and the intensity of RDF, the worm autofluorescence can potentially bias the results. However, based on our observations (Figure S1), the contribution of the worm autofluorescence (from either the GFP or RFP species present in the intestine of the worm) to the RFP signal is dwarfed by the bacteria-derived RFP signal and thus, in practice, the worm autofluorescence can be safely ignored.

## 4. Results

**Figure 6:**
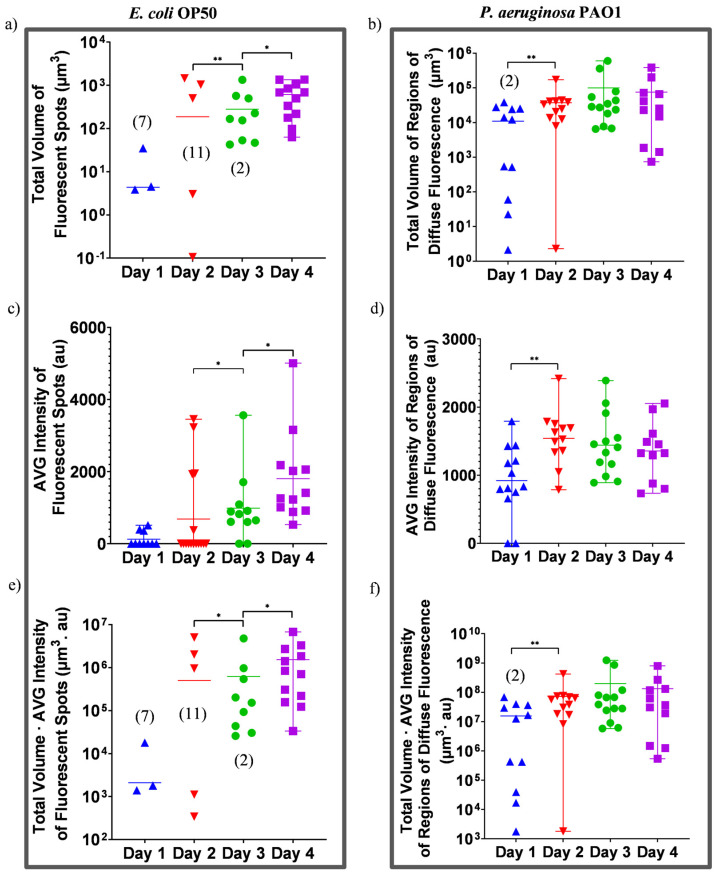
Single-worm-resolution analysis of bacterial load in adult worms that have fed on RFP-labeled bacteria for 1, 2, 3 and 4 days. Each data point is calculated for a single worm. The horizontal bars denote the average value and the error bars (where applicable) show the range of the data. The number of worms devoid of bacteria on each day is mentioned inside the plot alongside the data points of the respective day (applicable to (**a**,**b**,**e**,**f**)). An AVG intensity of zero indicates worms whose intestines are devoid of bacteria (applicable to (**c**,**d**)). (**a**) The total volume of fluorescent spots, (**c**) the background-corrected average intensity of fluorescent spots and (**e**) the multiplication of a and c, which is proportional to the total number of intact bacteria. (**a**,**c**,**e**) are obtained from fluorescent *E. coli*-OP50-fed worms with worm sample sizes of 10, 16, 11 and 12, for day 1 to day 4, respectively. (**b**) The total volume of RDF, (**d**) the average intensity of RDF and (**f**) the multiplication of b and d, which is proportional to the SIDM. (**b**,**d**,**f**) are obtained from fluorescent *P. aeruginosa-*PAO1-fed worms with worm sample sizes of 13, 12, 13 and 11 for day 1 to day 4, respectively. * *p* ≤ 0.05, ** *p* ≤ 0.01.

The image analysis by IMARIS allows us to analyze the intestinal bacterial content with single-worm resolution. In the case of worms that have fed on RFP-labeled *E. coli* OP50, IMARIS provides datasheets containing the volumes and the average intensities of every fluorescent spot in each worm. These datasheets are then imported into MATLAB software to be processed using basic arithmetic operations. For each worm, we determine the total volumes of the fluorescent spots. Additionally, we apply a background correction to the average intensities of the fluorescent spots by subtracting from them the background noise intensity. Afterward, a volume-weighted average of the background-corrected average intensities of all the fluorescent spots in each worm (referred to as “AVG Intensity”) is calculated. Thus, the bacterial load in every worm is proportional to the multiplication of the total volume and the “AVG Intensity” of the fluorescent spots. In the case of worms that have fed on RFP-labeled *P. aeruginosa* PAO1, IMARIS provides datasheets containing the volumes and the average intensities of the RDF in each worm that are similarly imported and processed using MATLAB. However, it must be remembered that in the case of RFP-labeled *E. coli-*OP50-fed worms, the bacterial load describes the total number of intact bacteria, while in the case of RFP-labeled *P. aeruginosa-*PAO1-fed worms, the bacterial load describes the Sum of Intact and Disrupted Bacteria (SIDB). Regardless, in both cases, the processed data are plotted in GraphPad, yielding Figure 6 where the single-worm-resolution analysis of intestinal bacterial load can be seen. 

Figure 6a shows the total volume of fluorescent spots in worms that have fed on RFP-labeled *E. coli* OP50. As there is a large variation in the data points, the use of log plot is necessary. As a result, no data points can be shown for worms whose intestines are free of bacteria (since log(0) is undefined). Instead, the number of worms devoid of bacteria on each day is mentioned inside the plot alongside the data points of the respective day. The absence of a number indicates the absence of worms devoid of bacteria on the respective day. This convention has been applied to plots in Figure 6a,b,e,f. We can observe in Figure 6a that the hindguts of almost all the worms that have fed on RFP-labeled *E. coli* OP50 are devoid of fluorescent spots and thus intact bacteria in the first two days. Nonetheless, there are three worms on the second day in which the total volume of the fluorescent spots is significantly large. This shows the importance of single-worm resolution analysis since considering the average of the total volume of the fluorescents spots alone would imply the presence of a significant volume of fluorescent spots in all the worms. On the third day, there is a sharp increase in the number of worms whose intestines contain significant volumes of fluorescent spots and, on day 4, the intestines of all the worms contain significant volumes of fluorescents spots. Similar observations can be made when the “AVG Intensity” of fluorescent spots is considered in Figure 6c. It should be noted that an AVG Intensity of zero indicates worms whose intestines are devoid of bacteria (similar convention also applies to Figure 6d). Additionally, we can further observe that the fluorescent spots grow in intensity between day 3 and 4, which shows that the density of intact bacteria within the bacterial spot is increasing. It must be mentioned that the worm-to-worm variation in the total volume of the fluorescence spots for each day (Figure 6a) is larger than that in the “AVG Intensity” of the fluorescent spots for the same day (Figure 6c), as evident from the use of the log axis in the former. Consequently, the plot representing the total number of intact bacteria within the intestine (Figure 6e), which is obtained by the multiplication of Figure 6a,c, mostly resembles the former. 

This means that the intestinal bacterial load increases through the spread of intact bacteria in the intestine, rather than the formation of very dense bacterial clusters. Overall, it can be understood that until day 2, most of the worms are devoid of any intact bacteria and it is on day 3 and 4 where a significant number of intact bacteria are found in the intestine of every worm. However, considering the upper and lower limits of bacterial load variation among the worms on day 3 and day 4, it seems that the number of intact bacteria is approaching a saturation limit. Figure 6b shows the volume of the RDF in the worms that have fed on RFP-labeled *P. aeruginosa* PAO1. It can be seen that on day 1, in about half of the worms, the total volume of the RDF is significantly large, and only in 25% of the worms is the total volume of the RDF very small (in fact, for two worms, the total volume of the RDF is zero but, due to using log axis, a value of 1 is assigned instead). On day 2, there is an increase in the total volume of RDF and through comparison of intrapopulation variations among day 2, 3 and 4, it can be inferred that the volume of the RDF has already reached a saturation limit. Figure 6d shows the AVG intensity of the RDF, representing the density of the sum of intact and disrupted bacteria, for different feeding periods. On day 1, except in two worms, the AVG intensity of the RDF is significant. On day 2, the AVG intensity of the RDF is higher than day 1 and has already reached a saturation limit as suggested by comparing intrapopulation variations among day 2, 3 and 4. Considering the use of a log axis in Figure 6b and a linear axis in Figure 6d, we can observe that for a worm population of each day, there is a larger intrapopulation variation in terms of the total volume of the RDF than in terms of “AVG Intensity”. Therefore, the plot representing the SIDB (Figure 6f), which is the result of multiplying Figure 6b,d, mostly resembles the former. This implies that the increase in bacterial load is through the spread of bacteria within the intestine rather than the formation of high-density but small RDFs. Based on Figure 6b, we can observe that on day 1, out of 13 worms, two worms have an SIDB of 0 (due to use of log scale, they are assigned a value of 1), 6 worms have a significantly high SIDM, while the rest of the worms are in between these two extremes. However, on day 2, all the worms have a significantly high SIDM and comparing their intrapopulation variation to those of worms on day 3 and 4 suggests that the SIDM has already reached a saturation limit after 2 days. 

We must stress that we have used two different methods for the quantification of bacterial load in RFP-labeled *E. coli-*OP50-fed worms and RFP-labeled *P. aeruginosa-*PAO1-fed worms. Moreover, in the former case, the bacterial load is proportional to the number of intact bacteria, while in the latter case, it is proportional to SIDB. Additionally, we have not measured the fluorescence signal intensity per unit of either (intact) bacteria. As a result, direct quantitative comparison between the two groups should be avoided. Nevertheless, it is reasonable to assume that when the intestine of the worm is filled with either of the two bacteria, as indicated by reaching a saturation limit in Figure 6e,f, the numbers of intact RFP-labeled *E. coli* OP50 and *P. aeruginosa* PAO1 bacteria present in the intestine are similar. This assumption allows us to compare the rate of bacterial colonization in the intestine of worms. In the case of RFP-labeled *P. aeruginosa*-fed worms, already on day 1, half of the worms contain a bacterial load similar to those of worms on day 4. However, in the case of RFP-labeled *E. coli-*OP50-fed worms, this takes place sometime between day 2 and day 3. The faster spread of RFP-labeled *P. aeruginosa* is also evident by the fact that after 2 days, the bacterial load reaches saturation, while for RFP-labeled *E. coli* OP50, the saturation can be thought to occur at the earliest on day 4. Therefore, in both cases, the intestinal bacterial load generally increases with the age of the worm, albeit more rapidly for worms fed on RFP-labeled *P. aeruginosa* PAO1. 

## 5. Discussion

*C. elegans* is a versatile model organism for studying bacterial pathogenesis and evolutionary conserved innate immunity pathways [11,18]. *E. coli* OP50, a non-pathogenic bacterium, is used as a food source for the general maintenance of *C. elegans* worms [14]. However, despite being a seemingly innocuous bacterium in the larval stages, as the worm ages past the L4 stage, due to the general decline in the functioning of the pharyngeal grinder and innate immunity, *E. coli* OP50 colonizes the intestine of the worm and thus becomes mildly pathogenic [9]. On the other hand, *P. aeruginosa* is a known pathogenic bacterium that is capable of killing the worm through different means, and in particular, intestinal colonization [16,20,21,22,23,24,25,26,27,28]. The average lifespan of worms fed on *P. aeruginosa* PAO1 is about 20% of those fed on *E. coli* OP50, which reflects the stark difference in the degree of pathogenicity [15,16]. 

Despite the extensive research on *P. aeruginosa* infection in *C. elegans* via intestinal colonization, there are no systematic studies on the feeding behavior and the digestion in infected worms. We do know, however, that within 8 h of feeding on *P. aeruginosa* PA14—a more virulent strain compared to PAO1 we used in our study—the intestine becomes distended, containing few intact bacteria that are the source of putative Outer Membrane Vesicles (OMVs) that are also present in the intestine [55]. Additionally, an unknown Extracellular Material (EM) covers the intact bacteria as well as the apical surface of the intestine’s brush border [55]. Unfortunately, it is not known whether the EM is produced by the bacteria or the worms [55]. After 24 h of feeding, the intestine becomes more distended, the numbers of intact bacteria and OMVs increase, and a thicker layer of EM covers the microvilli, which still retain their normal length [55]. At 48 h of feeding, the intestine becomes even more distended, and the increased number of intact bacteria are mostly separated from the now shortened microvilli by a thick layer of EM and intercellular invasion by the bacteria can be seen [55]. A similar pathogenesis has been observed in worms feeding on *P. aeruginosa* PAO1 [56] but worms fed on *E. coli* showed no such signs [55]. 

Whether and how the symptoms seen in *P. aeruginosa*-fed worms are related to the accumulation of disrupted bacteria we observed in Figure 4c is not established. It has been previously reported that worms fed on *P. aeruginosa* PAO1 have shorter body lengths compared to those fed on *E. coli* OP50 [57], indicative of a reduced metabolism. Probably, feeding on *P. aeruginosa* PAO1 reduces the rate of bacterial material absorption and/or defecation in the worms, which then results in the accumulation of nutrients/waste originating from disrupted bacteria in the gut. This hypothesis can be evaluated in future works, by observing how the time gap between the last feeding event and the fixation of the worms affects the pattern of the bacteria-derived fluorescence signals. In the present study, the time gap was 1 h, which was sufficient for *E. coli*-OP50-fed worms to fully digest the bacteria but not enough for *P. aeruginosa*-fed worms. Imaging the worms that are fixed with different time gaps (e.g., 15, 120 and 180 min) can eventually reveal the dependency of the digestion dynamics on the bacteria used as the food source. 

Previous studies on worms fed on either *E. coli* OP50 or *Salmonella typhimurium* (*S. typhimurium*) SL1344 show that the number of colony-forming bacteria in the intestine generally increases with the age of the worm until it reaches a saturation limit, albeit earlier in the case of the more pathogenic *S. typhimurium* SL1344 [15,18]. We obtained similar results and found that the bacterial load in worms fed on RFP-labeled *E. coli* OP50 seem to approach a saturation limit on day 4, while for those fed on RFP-labeled *P. aeruginosa* PAO1, the saturation limit is reached on day 2. This observation can be at least partially explained, as we alluded to earlier, by the reduced metabolism in the worms fed on *P. aeruginosa* PAO1.

Even though we used a simple microfluidic device in this work, the methods described in this work can be readily implemented in other types of microfluidic devices. Our group has already developed automated image and video processing techniques that can be used for in vivo extraction of phenotypes such as area, length, egg release rate and motility from the worms that are studied via microfluidic devices [40]. As discussed earlier, phenotypes such as length and area can help clarify the impact of bacterial food source on the development of the worm. Furthermore, our group has also reported devices dedicated to studying bacterial load dynamics and digestion [45,46]. The high-resolution imaging of the gut bacteria afforded by the methods described here can extend the capabilities of such devices and thus provide a better understanding of the interactions between bacteria and the worm.

## 6. Conclusions

We have demonstrated the potential of on-chip high-resolution z-stack fluorescence imaging and advanced image analysis for elucidating the bacterial food digestion and accumulation in *C. elegans* worms. We discovered that bacteria-derived fluorescence signals in the intestines of the worms fed on RFP-labeled *P. aeruginosa* PAO1 uniquely included a diffuse fluorescent background in addition to individual fluorescent spots that are also present in worms that were fed on RFP-labeled *E. coli* OP50. This observation indicates an intestinal accumulation of disrupted bacteria exclusive to RFP-labeled *P. aeruginosa-*PAO1-fed worms, which, when understood in the context of previous studies, hints at a reduced metabolism in such worms compared to those fed on RFP-labeled *E. coli* OP50. Furthermore, our single-worm resolution analysis showed that with both diets, the bacterial load in the intestine generally increases with age until it reaches a saturation limit. However, in the case of RFP-labeled *P. aeruginosa-*PAO1-fed worms, this limit was reached in 2 days compared to 4 days for RFP-labeled *E. coli* OP50, which further suggests a reduced metabolism in the former worms. The methods described here can readily be used to complement the scopes of existing microfluidic technologies.

## Data Availability

The data presented in this study are available in the supplementary material here.

## Supplementary Materials

The following supporting information can be downloaded at: https://www.mdpi.com/article/10.3390/mi14071386/s1, Excel datasheet of the bacterial load analysis in RFP-labeled *P. aeruginosa-*PAO1-fed worms and RFP-labeled *E. coli*-OP50-fed worms and the analysis of autofluorescence in RFP-labeled *P. aeruginosa-*PAO1-fed worms. Three-dimensional-rotating videos of Figure 4 and Figure 5 as viewed in IMARIS. Figure S1: High-resolution fluorescence images acquired in the RFP channel of representative fixed worms that have fed on RFP-expressing *P. aeruginosa* PAO1 for different durations. The ratio of the background-corrected average signal intensity in the intestinal lumen (as indicated by the yellow ribbon) to the background-corrected average signal intensity of an area outside of the intestinal lumen (as shown by blue rectangle) is equal to (a) 14, (b) 26, (c) 52 and (d) 30. The details of the calculations can be found in the excel datasheet provided.
