# Supplementary material for: Difference in Intestine Content of Caenorhabditis elegans When Fed on Non-Pathogenic or Pathogenic Bacteria
Source: Micromachines (Basel). 2023 Jul 7;14(7):1386. doi: 10.3390/mi14071386 (PMC10384281; doi:10.3390/mi14071386)
Supplement: Supplementary file 1 [file micromachines-14-01386-s001.zip › micromachines-2403028-supplementary.pdf]

Supplementary information

Difference in intestine content of *Caenorhabditis elegans* when fed on non-pathogenic or pathogenic bacteria

Farzad Rezaeianaran* and Martin A. M. Gijs

Laboratory of Microsystems, Ecole Polytechnique Fédérale de Lausanne, CH-1015 Lausanne, Switzerland.

*Author to whom correspondence should be addressed

E-mail: rezaeianaran.farzad@epfl.ch

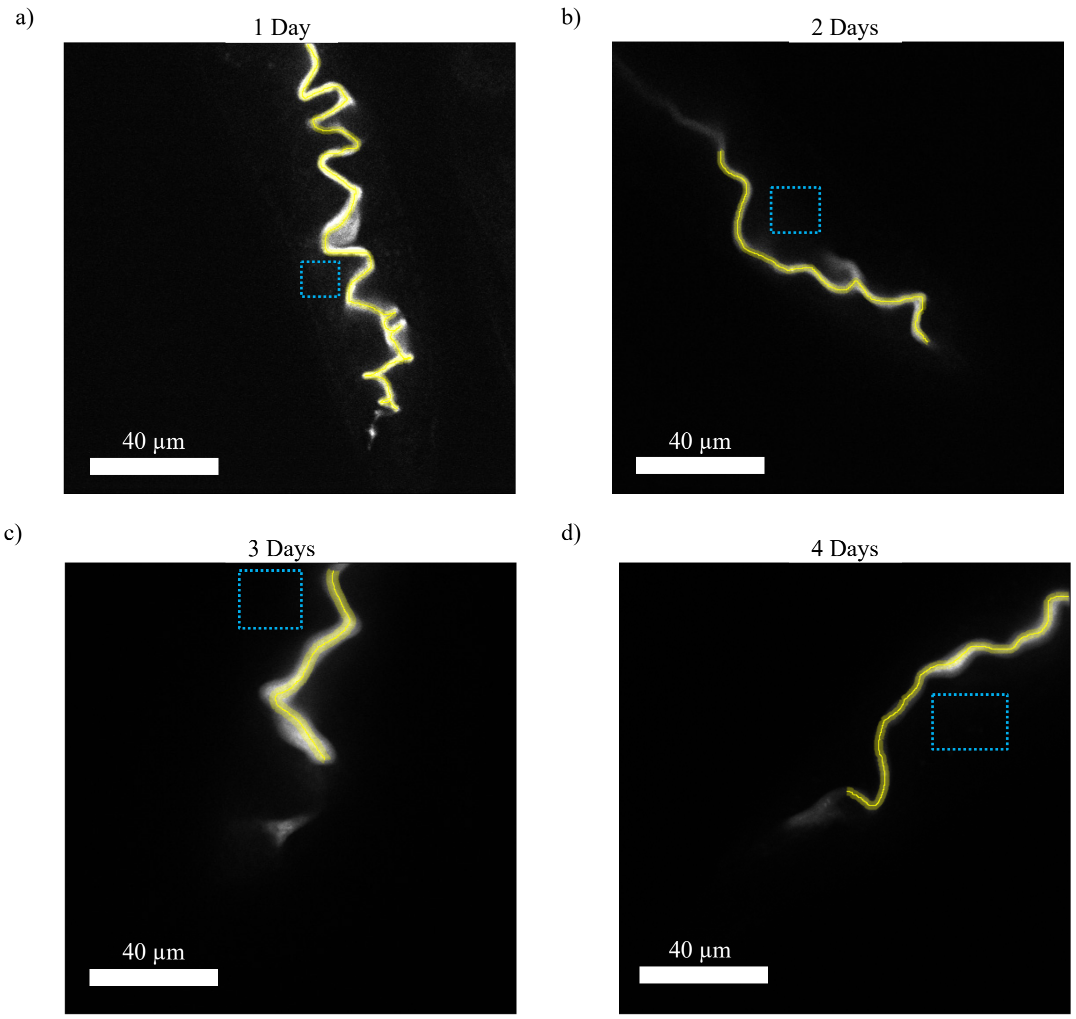

Figure S1. High-resolution fluorescence images acquired in the RFP channel of representative fixed worms that have fed on RFP-expressing *P. aeruginosa* PAO1 for different durations. The ratio of the background-corrected average signal intensity in the intestinal lumen (as indicated by the yellow ribbon) to the background-corrected average signal intensity of an area outside of the intestinal lumen (as shown by blue rectangle) is equal to (a) 14, (b) 26, (c) 52 and (d) 30. The details of the calculations can be found in the excel datasheet provided.

Datasheet: Excel datasheet containing the bacterial load analysis in RFP-labelled *P. aeruginosa* PAO1-fed worms, RFP-labelled *E. coli* OP50-fed worms and the analysis of autofluorescence in RFP-labelled *P. aeruginosa* PAO1-fed worms.

Movie_Figure4b: A rotating movie showing a 3D view of Figure 4b

Movie_Figure4b_U: A rotating movie showing a 3D view of Figure 4b

Movie_Figure 4d: A rotating movie showing a 3D view of Figure 4d

Movie_Figure 5a: A rotating movie showing a 3D view of Figure 5a

Movie_Figure5b: A rotating movie showing a 3D view of Figure 5b

Movie_Figure 5d: A rotating movie showing a 3D view of Figure 5d
